# Wearables and the Quantified Self: Systematic Benchmarking of Physiological Sensors

**DOI:** 10.3390/s19204448

**Published:** 2019-10-14

**Authors:** Günther Sagl, Bernd Resch, Andreas Petutschnig, Kalliopi Kyriakou, Michael Liedlgruber, Frank H. Wilhelm

**Affiliations:** 1 Department of Geoinformatics—Z_GIS, University of Salzburg, 5020 Salzburg, Austria; Andreas.Petutschnig@sbg.ac.at (A.P.); Kalliopi.Kyriakou@sbg.ac.at (K.K.); 2 Center for Geographic Analysis, Harvard University, Cambridge, MA 02138, USA; 3 Department of Psychology, University of Salzburg, 5020 Salzburg, Austria; Michael.Liedlgruber@sbg.ac.at (M.L.); Frank.Wilhelm@sbg.ac.at (F.H.W.)

**Keywords:** wearable sensors, psychophysiology, sensor data analysis, time series analysis, signal analysis, similarity measures, correlation statistics, quantitative analysis, benchmarking

## Abstract

Wearable sensors are increasingly used in research, as well as for personal and private purposes. A variety of scientific studies are based on physiological measurements from such rather low-cost wearables. That said, how accurate are such measurements compared to measurements from well-calibrated, high-quality laboratory equipment used in psychological and medical research? The answer to this question, undoubtedly impacts the reliability of a study’s results. In this paper, we demonstrate an approach to quantify the accuracy of low-cost wearables in comparison to high-quality laboratory sensors. We therefore developed a benchmark framework for physiological sensors that covers the entire workflow from sensor data acquisition to the computation and interpretation of diverse correlation and similarity metrics. We evaluated this framework based on a study with 18 participants. Each participant was equipped with one high-quality laboratory sensor and two wearables. These three sensors simultaneously measured the physiological parameters such as heart rate and galvanic skin response, while the participant was cycling on an ergometer following a predefined routine. The results of our benchmarking show that cardiovascular parameters (heart rate, inter-beat interval, heart rate variability) yield very high correlations and similarities. Measurement of galvanic skin response, which is a more delicate undertaking, resulted in lower, but still reasonable correlations and similarities. We conclude that the benchmarked wearables provide physiological measurements such as heart rate and inter-beat interval with an accuracy close to that of the professional high-end sensor, but the accuracy varies more for other parameters, such as galvanic skin response.

## 1. Introduction

In the last decade, the body of literature about physiological sensing and deriving emotions from physiological parameters has grown significantly. One reason for this is the rapid increase in variety of affordable wearable sensors that measure a broad range of physiological parameters such as heart rate, galvanic skin response, skin temperature, and others. With this increase, the “Quantified Self” community that promotes the idea of 24/7 tracking and monitoring has been growing significantly [1,2,3].

These new low-cost wearables are increasingly used in scientific studies in a variety of areas like health research, well-being assessment, disaster management, emotion information extraction and spatial emotion analysis, and stress detection [4,5,6,7,8,9,10,11,12,13]. However, some research efforts have used wearable physiological sensors without prior investigation of the sensor’s exact quality parameters, i.e., how accurately a sensor actually measures a given parameter or how reliable a sensor is in producing continuously high-quality measurement results.

Understanding a sensor’s quality and accuracy is critical because the research results may otherwise be unreliable: while traditional professional wired sensor devices, which have been used for some time in laboratory and ambulatory studies in the fields of psychological and medical research, are proven to be highly accurate, most wearable sensors used in previous studies are not. In fact, most of them are not medically and/or electronically certified, which compromises the reliability of the measurement results. However, recently, some wearable sensors have been released that are certified and comply with a number of international standards (sensor technology, wireless communication, data transmission, etc.), which makes them a viable alternative to traditional wired equipment.

In the context of this research, we aim to investigate the measurement quality of two wearable sensor devices, namely the Zephyr BioHarness 3 and the Empatica E4, by comparing their measurements to those of calibrated laboratory sensors. Concretely, we are interested in the similarity and correlation of univariate time series from two different sensors that measure the same physiological parameters at the same time on the same participant. To evaluate the accuracy of the low-cost sensors, we perform benchmark testing between low-cost sensors against high-quality and well-calibrated sensors that act as the trusted gold standard. The second aim of this research is to detect and quantify relationships and dependencies between pairs of the same and different physiological parameters measured by different sensors. Our study assesses the parameters heart rate (HR), inter-beat interval (IBI), and galvanic skin response (GSR).

The remaining part of the paper is structured as follows. In Section 2, we provide a concise summary of related work regarding sensor benchmarking, followed by an overview of the physiological parameters of interest and the sensors used for this research (Section 3). The benchmarking methodology is presented in Section 4, where we also explain the entire workflow from sensor data acquisition to the analysis results. Section 5 descriptively illustrates the results, including a variety of statistical visualisations of similarity and correlation patterns. Finally, we discuss the results obtained and close the paper with our core conclusions.

## 2. Sensor Benchmark Methods—Related Work

The analysis of physiological signals from wearable sensors in order to better understand the human emotional response to the immediate surroundings has been investigated for several years. In recent years, a variety of affordable wearable sensors that measure well-established physiological parameters, such as heart rate and galvanic skin response, has reached the market. As a logical consequence—and as already mentioned in the introduction—the “Quantified Self” community is growing faster than ever, and inspiring scientific research, especially related to emotion and stress detection [5,7,8,9,14,15,16,17]. In any case, the basis for any further advanced analyses is adequate data quality in terms of accuracy, reliability, and validity [7,9,18]. However, scientific literature about the similarity and correlation of the measurements from such affordable wearables compared to those from well-calibrated and high-quality sensors from scientific laboratories is rare.

### 2.1. Similarity Measures

Generally speaking, the term ‘similarity’ is not rigorously mathematically defined. A variety of similarity measure families exist, for instance, distance-based (e.g., Euclidean distance), feature-based (e.g., Fourier coefficients), model-based (e.g., autoregressive), and elastic measures such as Dynamic Time Warping (DTW) and Edit Distance on Real sequence EDR [19,20,21,22]. A comprehensive review, however, is out of the scope of this paper—the interested reader may refer to [19,20,23,24], among other work.

In this research, we go beyond global measures and linear models to assess similarity. To uncover local similarity characteristics of time series, we thus follow a moving window approach combined with more informative distance metrics. Elastic measures, such as DTW and the Fréchet distance, allow for a one-to-many comparison of time series elements, while so-called “Lock-Step” measures, such as Euclidian and Manhattan distance, only allow comparison of fixed pairs, making them very sensitive to local time-shifts and noise [23].

DTW temporally aligns two time series using the shortest path in a distance matrix, i.e., the path with the minimal global warping distance [25,26], thereby finding the most representative distance of the overall difference [20]. However, a comprehensive experimental comparison of representation methods and distance measures of time series reveals inconsistencies and even contradictions in the observations reported in individual studies [23]. An important consequence of this is that experimental results cannot be generalised without critically reviewing the assumptions made for a particular research context and study design. As concluded in [23], “there is no clear evidence that one similarity measure exists that is superior to others in the literature in terms of accuracy. While some similarity measures are more effective on certain data sets, they are usually inferior on some other data sets” (p. 297). The DTW distance outperforms Euclidian distance in a variety of studies [27]. Other types of measures are “Edit measures” and “Threshold measures”. The former type includes, for instance, Longest Common Sub-Sequence LCSS, Edit Distance on Real sequence EDR and Edit Distance with Real Penalty ERP. The latter type includes Tightness of Lower Bounds TLB. The accuracy of the aforementioned other types is close to the accuracy of DTW, but DTW is much simpler [23,28]. We thus concluded to use DTW to assess the temporal similarity of the physiological time series.

To assess the geometric shape of a curve or curve segment, other distance measures, such as the Fréchet distance [29], can be used [30,31,32]. “The Fréchet distance is typically explained as the relationship between a person and a dog connected by a leash walking along the two curves and trying to keep the leash as short as possible. The maximum length the leash reaches is the value of the Fréchet distance” [33] (p. 7). We thus use the Fréchet distance to assess the geometric similarity of time series of sensor measurements of the same physiological parameter (e.g., GSR) on the same participant at the same time but with different sensors.

### 2.2. Correlation Statistics

The correlation of time series has been investigated for decades, in diverse fields. Herein, our focus on time series correlation is twofold: first, the correlation between equal-type physiological parameters measured by different sensors at the same time on the same participant in order to quantify differences between low-cost and un-calibrated sensors versus high-end and calibrated laboratory sensor equipment; second, the correlation between physiological parameters of different types, for instance, IBI and GSR, to explore potentially hidden relationships.

According to [34], the Pearson’s correlation coefficient is the most robust metric when measuring the similarity in physiological time series—where robustness is understood as insensitivity to small variations. However, Pearson’s r is highly sensitive to outliers and only considers linear relationships. Spearman’s rank correlation coefficient (rho) is—as the name says—based on the rank of the values rather than on the values themselves; thus, it measures monotonicity rather than linearity. Therefore, using Spearman’s rho to measure the strength of the associations between two variables leaves room for interpretation [35].

The human cardiovascular system and the autonomic nervous system are highly non-linear systems. In order to explore possible underlying non-linear interactions in the relationship between different physiological parameter, we herein, use the Maximum Information Coefficient (MIC) [36,37]. Several studies show the possibility of gaining new insights into such non-linear interactions when applying the MIC, for instance, in the interactions between neural and respiratory dynamics [38].

Further, one method to assess the temporal lag (or lead) between pairs of time series is the cross-correlation function in the time domain [39]. To get meaningful cross-correlation results, the time series need to be stationary, i.e., have a constant mean and variance. Time series stationarity can be tested using, for instance, the Augmented Dickey-Fuller test [40]. Unless the time series is stationary, it needs to be differenced and tested for stationarity.

## 3. Physiological Parameter of Interest and Sensors used for Benchmarking

Herein, we describe the physiological parameters we investigated, and the sensors used to measure them. We investigated three sensors and four physiological parameters (Table 1):

- HR: heart rate, i.e., heartbeat frequency, unit: beats per minute
- IBI: inter beat interval, also known as the RR interval, i.e., the time between two R-peaks in the ECG’s QRS complex, unit: milliseconds
- ECG: electrocardiogram, i.e., electrical activity of the heart, unit: millivolt
- GSR: galvanic skin response, i.e., the level of electric conductance of the skin, unit: microSiemens [μS]

### 3.1. VarioPort

The VarioPort (http://www.bisigma.de) is a small, lightweight, and highly flexible recording system that is used for multi-channel physiology recordings in laboratory and ambulatory setups. The standard version of the device can record up to 16 signals from connected pre-amplifiers (e.g., electromyography, electrocardiography, electrodermal activity, or respiration). The device has two built-in marker buttons that can be used to signal certain events occurring over time, resulting in an additional channel of data. We used these buttons to mark changes in the activity phases (for details refer to Section 4.1). Available sensors are either wire-connected to the device or are directly integrated into the device. The recorded data are stored on an SD card inside the device. The VarioPort allows setting different sampling rates for different channels, thus effectively reducing the required storage, especially in case of slowly changing signals (e.g., such as the skin conductance). For rapidly changing signals, such as ECG, sampling rates of up to 1024 Hz can be set. Since the VarioPort is the platform used for scientific studies at our Psychology Department, we used it as the gold standard in our benchmarking. In the remaining part of the paper, the VarioPort sensor is called VP.

### 3.2. Zephyr BioHarness 3

The Zephyr BioHarness 3 (https://www.zephyranywhere.com/) is a multivariable physiological monitoring device with a chest belt sensor that measures a wide variety of physiological parameters. The BH is a certified medical product (FDA Class II). Due to its design as a chest belt, the BioHarness 3 can measure ECG, RR intervals, respiration frequency, and other parameters such as 3D acceleration on a single sensor platform. Furthermore, parameters such as heart rate can be derived from the directly measured parameter, for instance, from ECG (HR within 0–240 BPM and an accuracy of ±1 BPM). The sampling rate for ECG is 250 Hz. The BH and the smartphone are connected wirelessly via Bluetooth. The raw data are accessible via a free SDK in binary format, and the device has been extensively tested and validated in practical applications [41,42]. In the remaining part of the paper, the Zephyr BioHarness 3 sensor is called BH.

### 3.3. Empatica E4

The Empatica E4 (https://www.empatica.com/research/e4/) is a wrist band sensor that measures HR and GSR, as well as other parameters. The E4 is medically certified according to CE Medical 93/42/EEC Directive, class 2a, FCC. The sampling rate for GSR is 4Hz and for IBI 64Hz. According to Gradl et al. [18], the E4 is a wearable sensor that has the potential to measure mental stress. The E4 allows access to the raw data via smartphones through a comprehensive SDK and a Bluetooth connection. In the remaining part of the paper, the Empatica E4 sensor is called E4.

## 4. Benchmark Method

### 4.1. Study Setup and Participants

Our study included 18 participants who were recruited via e-mail. The test group comprised nine females and nine males in an age range of 24 to 40 years. All test persons were physically in decent shape and did not suffer from any illness at the time of the study.

The study was carried out at the University of Salzburg’s Department of Psychology. After the study leaders attached the sensors, the participants were instructed to sit on an ergometer and follow the following routine:

- Resting phase: 5 min of rested seating on the ergometer, not performing any physical activity; used as a calibration phase for the measurements
- Cycling phase: 10 min of cycling at a constant 50 rpm; stepwise increase of physical load (5 steps of 2 min each, during which the resistance/power of the ergometer was increased from 35–65–100–133–165 W)
- Cool down phase: 5 min cool down, rested seating like in the resting phase

Participants were told not to interact with other people in the room, to focus on their task, and not to perform any physical activity other than as instructed. This was observed by the study leaders. For each ‘run’, two test persons were doing the lab study in parallel next to each other. Before commencing the actual exercise, we checked that all sensors were well positioned according to the participants’ individual body shape. Additionally, we used surgical tape as necessary to hold the devices in place to make sure that we receive plausible measurements. We conducted these checks for each participant individually. All participants were aware of the aim of this research, and we obtained informed consent from all participants prior to commencement of the study.

### 4.2. Data Acquisition

**Figure 1:**
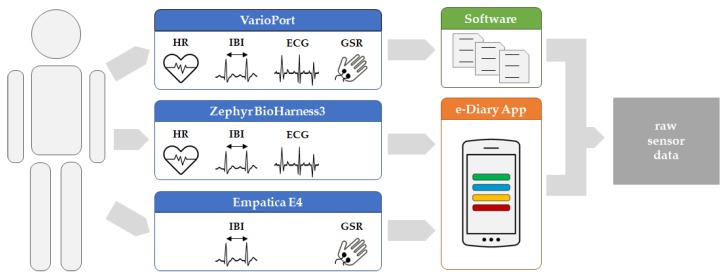
Data acquisition workflow—from the human participant (**left**) to raw sensor data (**right**).

The basic data acquisition workflow is illustrated in Figure 1. Each participant was equipped with diverse sensors to measure the physiological parameter using different platforms, namely VarioPort (VP), Zephyr BioHarness3 (BH), and Empatica E4 (E4). For each run, which refers to a participant exercising while their physiological parameters are measured, the raw sensor data are either stored in an SQLite database directly on the smartphone, or as files in a proprietary format on an SD card. The measurements from VP were extracted to flat files using the Software ANSLAB [43]. In contrast, the measurements from BH and E4 were sent to and fused by the e-Diary App into an SQLite database. The “raw sensor data” serves as input for the pre-processing, which is necessary to prepare the data for further analyses. The e-Diary App is herein purely used for sensor data collection and data management. During real-world field studies, however, the e-Diary App collects additional data such as GPS positions and contextual user feedback used for ground-truthing, thereby enabling the investigation of moments of stress in a spatio-temporal and contextual manner [4,44].

### 4.3. Data Pre-Processing

**Figure 2:**
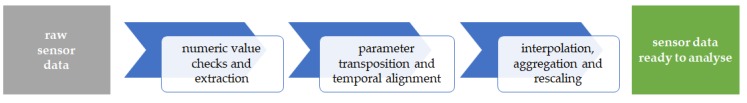
Data pre-processing phase—from raw sensor data (**left**) to sensor data ready to analyse (**right**).

The “raw sensor data” from the previous step serves as input for the data pre-processing phase, which is illustrated in Figure 2.

This pre-processing phase consists of three main steps:

1. Extracting numeric values from differently encoded strings of values:

Data in the SQLite DB are stored in 1 s intervals in different formats due to various sampling rates of each of the sensors measuring different physiological parameters. For instance, the Empatica E4 measures GSR at a sampling rate of 4 Hz, while the VarioPort measures GSR at a sampling rate of 25 Hz.

The result is a table with sensor values where each single measurement has a correct timestamp.

2. Transposition of parameters and temporal alignment of measurements:

First, the vertical parameter structure (one row consists of a timestamp and a single sensor measurement, the next row consists of the same timestamp and with another single sensor measurement) needs to be transposed to a horizontal structure (a common timestamp and individual values as columns: one row consists of a timestamp and all sensor measurements that occurred at that timestamp).

Second, the irregular timestamps of all measurements are aligned to the millisecond in order to ensure the best possible time matching to the sensors’ synchronized time. Since a 1 millisecond resolution is below the original sampling period, the measurements are aggregated depending on the parameters.

The result is a regular multivariate time series with 10 or 100 millisecond resolutions where some parameters at some timestamps may be missing values while other parameters are averaged within the given millisecond interval.

3. Interpolation, moving average and rescaling:

To fill missing values introduced by the temporal alignment in step 2, we applied spline interpolation, because it tends to greatly reduce oscillation by taking into account data points before and after the gap to be interpolated for a continuous representation [45]. In addition to the raw data, we calculated a moving averaged version with a window of ±5 s to eliminate high local variations. For the correlation analysis of same type parameters and exploratory plots, we keep the original scaling of individual time series to identify potential offsets of measurements. For the similarity analysis, however, we rescale the measurements of individual time series from minimum and maximum to 0 and 1 in order to compare similarity distance metrics.

The data pre-processing was mainly carried out directly in the database using SQL and Java.

### 4.4. Statistical Signal Analysis—Time Series Correlation and Similarity Analysis

**Figure 3:**
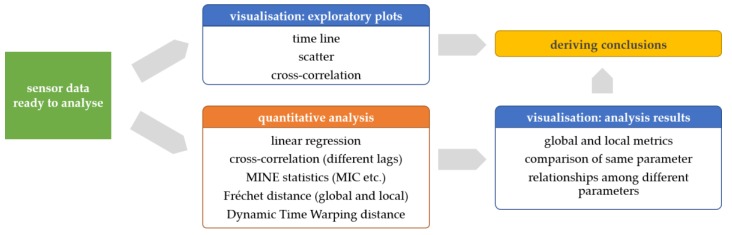
Data analysis workflow—from sensor data ready to analyse (**left**) to visualizations of exploratory plots and quantitative analysis results to deriving conclusions (**right**).

This sub-section illustrates how we assessed the correlations and similarities between time series of the same physiological parameters measured by different sensor platforms. Additionally, for some selected statistics, such as the MIC, we also run the analysis between different physiological parameters in order to explore potentially unknown relationships. The basic analytical workflow is shown in Figure 3. Note that we use the original signal scaling for exploratory plots, linear regression, and cross-correlation, while we use the rescaled signal (minimum → 0…maximum → 1) to get similarity measures such as Fréchet and DTW distance.

Of the complete time series derived from the pre-processing workflow (see Section 4.2), we focused on the following physiological parameters: HR, GSR, IBI, ECG. Additionally, from the ECG signal, we again derived the IBI and the Complex Demodulation amplitudes for the following frequency bands to estimate the heart rate variability [46]:

- Very low frequency VLF (0.025–0.07 Hz)
- Low frequency LF (0.07–0.14 Hz)
- High frequency HF (0.14–0.5 Hz)

In order to quantify pairwise correlations and similarities, we focused on:

- The linear regression coefficient of determination R^2^, to assess the fit of pairwise timer series to a linear model [23]
- Cross-correlation, to assess temporal shifts [47]
- Maximal Information-based Nonparametric Exploration MINE statistics, in particular, the MIC, to assess functional associations, and MIC-R^2^ to assess non-linear associations [36,48]
- Fréchet distance, to explore geometric similarity [30,49]
- DTW distance, to explore temporal similarity [26,28]

Signal analysis and most plots were done using the statistical computing software R, while some other plots were produced with the data visualisation software Tableau Desktop.

## 5. Results

For each of the 18 participants, we captured 15 parameters either measured directly or derived from ECG. For all these parameters, we also computed a moving average for an outlier-smoothed version of the same signal in order to get a better understanding of the signal’s overall robustness and reliability. Additionally, we computed low/high-pass filtered versions of the GSR signals, as well as the complex demodulation amplitudes from the ECG signals. For each participant, we analysed 22 pairs of physiological parameters of the same type regarding similarity (e.g., heart rate from BioHarness sensor and heart rate from VarioPort sensor), and another 136 pairs of parameters of different type regarding correlations (e.g., heart rate from BioHarness sensor and galvanic skin response from VarioPort sensor).

Since there are many different parameters, we defined a naming convention that includes the physiological parameter of interest, the platform used to measure it, plus an indication of whether a time series is a moving averaged and/or a filtered version. For the naming of these parameters, we use the following notation:

For direct measurements:

*<parameter> <platform>* [*filt.*] [(*mv. avg*)]

where *parameter* can be GSR, HR, or IBI and *platform* can be BH, E4, or VP; *(mv. avg.)* indicates that this is the moving averaged version; *filt.* indicates that a first order high-pass (0.05 Hz) and first order low-pass (0.5 Hz) Butterworth filter has been applied to the original signal (this filter setting is used for further analysis to identify moments of stress [4,11]; however, this is not within the scope of this paper).

example: *GSR: VP (mv. avg.)* refers to the moving averaged version of galvanic skin response measured by VarioPort

For derived measurements:

<derived parameter> from <original parameter> <platform> [(mv. avg.)]

where *derived parameter* can be HF, IBI, LF, VLF, *original parameter* can be ECG, and *platform* can be BH, or VP; *(mv. avg.)* indicates that this is the moving averaged version example: *IBI: from ECG BH* refers to the inter beat interval derived from the electrocardiogram measured by BioHarness

The following subsections are structured according to Figure 3. We use two representative time series, one HR and one GSR, as examples to guide the reader through the high number of physiological parameters investigated herein. These two examples are cross-referenced between several figures and thus provide views on the same data from different perspectives, thereby fostering the consolidation of a more holistic picture.

### 5.1. Visualisation: Exploratory Plots

The aim of the exploratory plots is to obtain a basic understanding of the temporal behaviour and the relationship of equal-type physiological parameters. For this first insight, we investigate three complementary plots that provide different views on the same data: a time series plot, a scatter plot, and a cross-correlation plot. These plots show two versions of the same parameter, namely a data-as-is version and moving averaged version. Note that for the cross-correlation plots the second parameter is used as the independent one. To illustrate the methodology and exemplary results, we only show representative sample plots, which highlight the characteristic patterns of about 80% of all plots. Overall, we produced more than 1000 plots based on unscaled and rescaled data.

**Figure 4:**
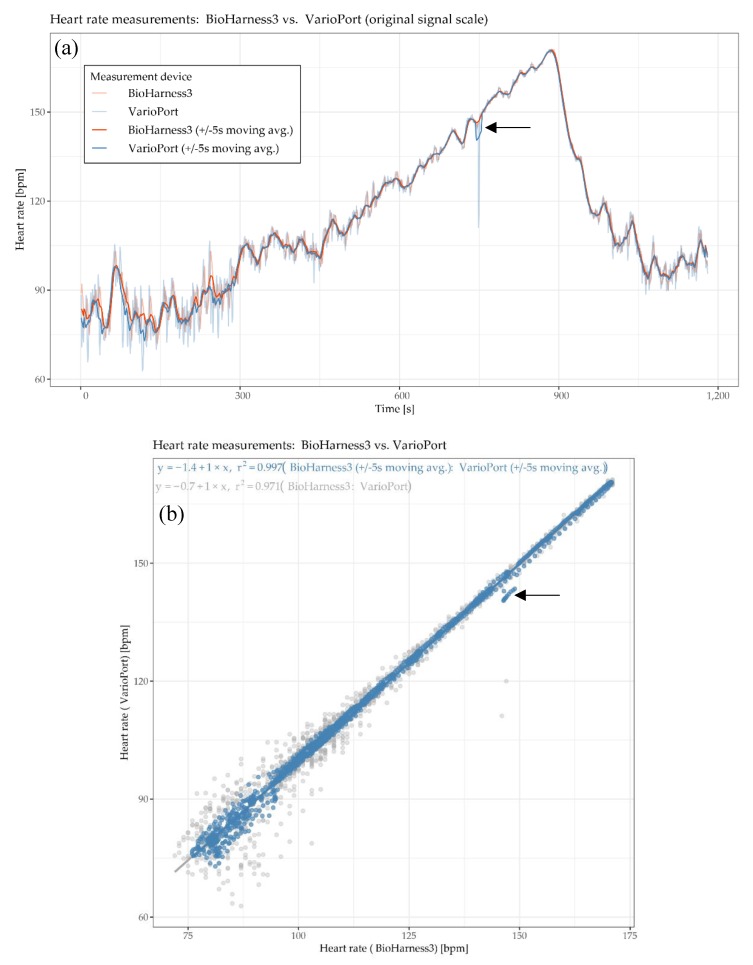

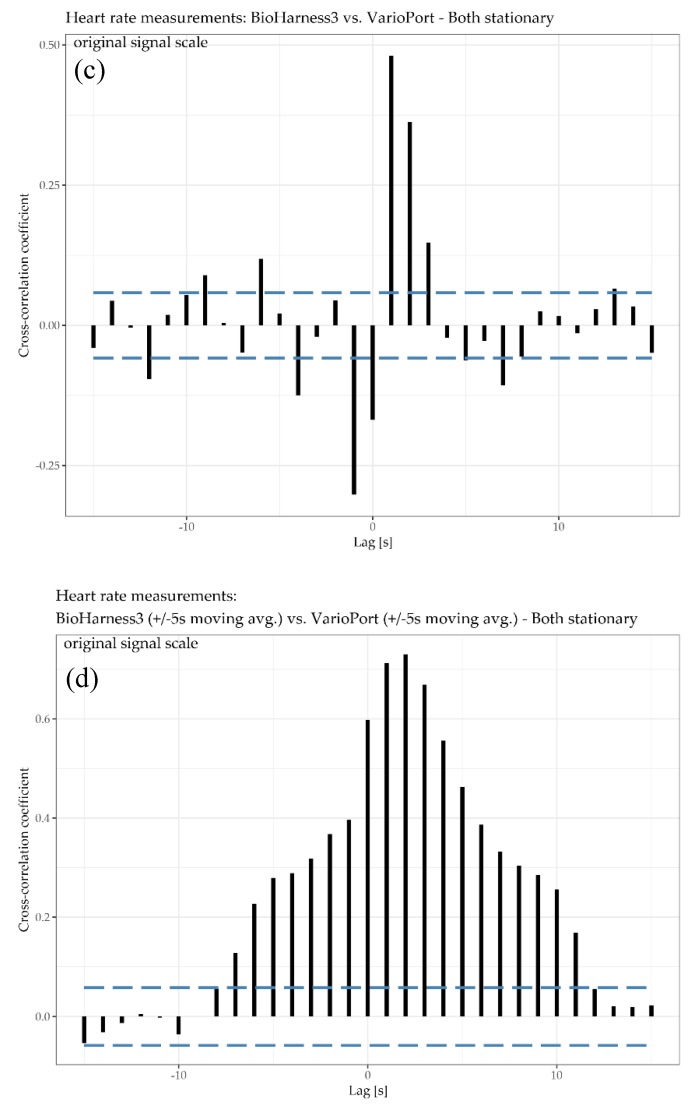
Participant RP 5–14: time plot (**a**), scatter plot (**b**), and cross-correlation plot (**c**), and cross-correlation plot of moving averages (**d**) of heart rate HR [beats per minute] measured by Bioharness3 BH sensor and VarioPort VP.

**Figure 5:**
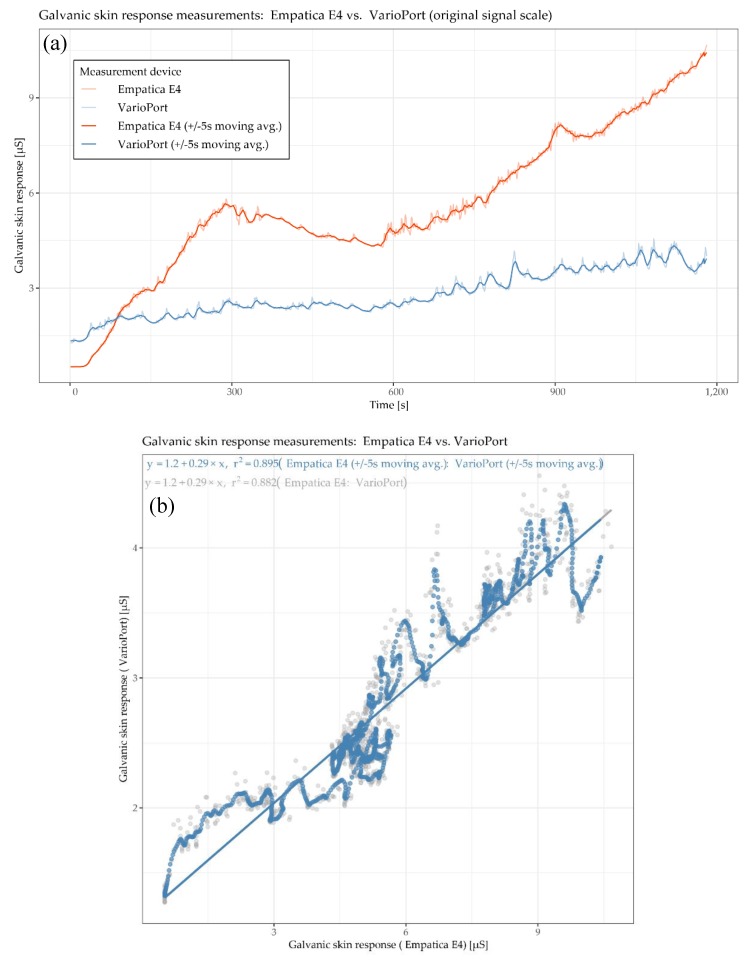

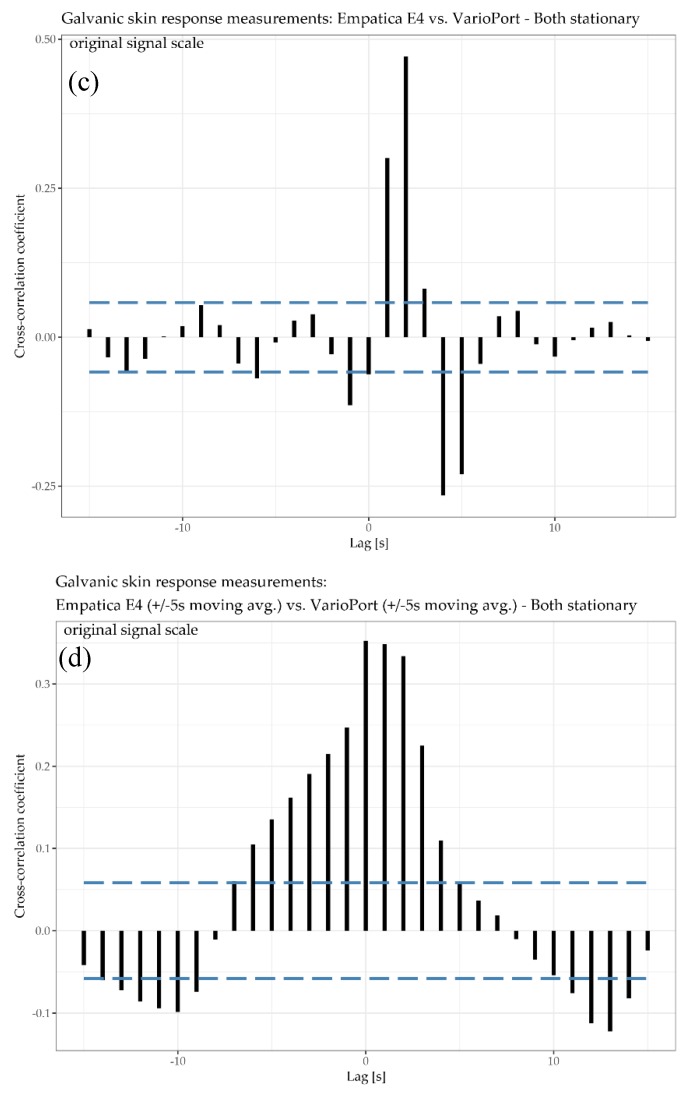
Participant RP 3–8: time plots (**a**), scatter plot (**b**), cross-correlation plot (**c**), and cross-correlation plot of moving averages (**d**) of galvanic skin response GSR measured by Empatica E4 and VarioPort.

Figure 4 and Figure 5 each show a time plot (a), a scatter plot (b), and a cross-correlation plot (c,d). The physiological parameter of interest is HR, measured by HB and VP. The HR time plot (Figure 4a) shows two highly similar, almost identical curves. The blue curve has an offset, which is maximal in the low range and converges to zero in the high range. The corresponding error term seems to include a reciprocal component: the higher the actual measurement, the lower the error. The HR scatter plot (Figure 4b) shows a high positive linear relationship with an R^2^ of 0.971 for raw data and 0.997 for the moving averaged data. This means that 97.1% and 99.7%, respectively, of the data’s total variance can be explained by a linear model. This plot also confirms what is seen in the time plot, namely that in the low range the residuals are higher than in the high range. Note that the higher residuals in red in the upper right quarter of the plot refer to the time plot at ~750 s, where the blue curve drops below the red curve (indicated by a black arrow in Figure 4a,b). The HR cross-correlation plots show the highest cross-correlation for the as-is version (Figure 4c) at a lag of 1 s, and the highest cross-correlation for the moving averaged version (Figure 4d) at a lag of 2 s. In other words, the local trend of the BH is lagging 1 and 2 s, respectively, “behind” the local trend of the VP on average.

In Figure 5, the physiological parameter of interest is GSR, measured by the E4 and the VP. Generally speaking, measuring GSR is, in comparison to HR, a delicate undertaking due to the measurement principle, which is solely based on the electrical conductivity of the skin. This conductivity is highly dependent on (1) the participant’s skin characteristics and (2) the contact between the sensor electrodes and the skin, especially during physical activity. Thus, these two factors can have a significant impact on the reliability, and thus on the comparability, of the sensor measurements. Additionally, the mounting of the sensors’ electrodes can differ as well. For instance, the VP electrodes need an isotonic electrolyte gel to ensure reliable measurements, while E4 does not require anything. The GSR time plot (Figure 5a) shows that the blue curve (VP) increases gradually. The red curve (E4) increases faster than the blue one and shows a local maximum after the 5-min warm-up phase (at around 300 s). This increase is followed by a decrease for another 5 min (until around 600 s). Aside from a little drop at around 900 s, the red curve increases until the end of the cool down phase. In general, the E4 seems to be more responsive to sweating associated with physical effort than the VP, which may be due to its lack of stabilizing isotonic electrolyte gel. The GSR scatter plot (Figure 5b) shows a positive correlation with an R^2^ of 0.882 for raw data and 0.896 for the moving averaged data. This means that 88.2% and 89.6%, respectively, of the data’s total variance can be explained by a linear model. The cross-correlation plots (Figure 5c) show the highest cross-correlation for the as-is version at lag of 2 s, and the highest cross-correlation for the moving averaged version at a lag of 1 s. In other words, the local trend of the E4 sensor is lagging 2 and 1 s, respectively, “behind” the local trend of the VP sensor on average.

### 5.2. Quantitative Analysis

The aim of the quantitative analysis is twofold: first, we assess the correlation and the similarity of equal-type parameters. Second, we assess potential associations in pairs of parameters of both equal-type and different types. In addition to global statistics, we also apply local measures to derive new information about the relationship between and among the different parameter pairs. This combination of global and local similarity and correlation measures on the individual level further enables a roll-up view on relationship patterns of physiological parameters among participants. Note that for similarity distance metrics, we rescaled measurements from min…max of the original scale to 0…1. For the correlation analyses we used the original values of the given parameter at the given range and the given unit in order to identify potential offsets.

#### 5.2.1. Linear Regression and Coefficient of Determination R^2^

**Figure 6:**
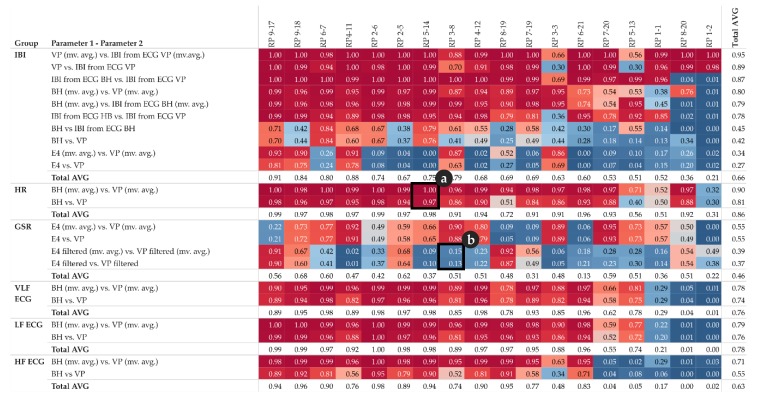
R^2^ matrix of pairs of parameter and participants; detail (**a**) complements Figure 4, detail (**b**) complements Figure 5; total average of individual pairs among participants is shown in the last column; total average of participants among individual parameter pairs is shown in the last row of each parameter group; colour: red indicates high correlation, blue indicates low correlation.

The first statistic of interest is the coefficient of determination R^2^, which quantifies the percentage of the variance of the two given parameters that can be explained by a linear regression model. In addition to the R^2^ of individual pairs of parameters, as shown in the scatter-plots (refer to Section 5.1, Figure 4b and Figure 5b), we now investigate all pairs among all participants and explore the corresponding R^2^ pattern. This pattern can be derived from the R^2^ matrix shown in Figure 6. Furthermore, for each group of parameters, e.g., all IBI related parameter, we calculate the total average per participant in order to get an impression of the impact of each participant’s individual overall measured activity (GSR base level, skin contact of electrodes, etc.).

In the upper half of the R^2^ matrix, the pairs of equal-type parameters, measured by different sensors (or derived from another signal of the same sensor) show a high linear relationship across the majority of the participants. This relationship also indicates that these parameters are rather robust from a measuring point of view. However, the matrix also shows some cases with no relationship at all, see for instance IBI derived from ECG BH (moving averaged version), and IBI derived from ECG VP (moving averaged version) at row three for participant RP 8-20 and RP 1-2. This may indicate that one of the sensors did not have proper contact between the electrodes and the skin and thus failed to collect valid data.

The matrix shows that GSR in general, and IBI measured by Empatica E4, tend to have rather low or even no correlation, while some participants demonstrate the exact opposite (compare instance RP 4-11 and RP 2-5).

Note that the R^2^ matrix in Figure 6 is organized as follows: for each group of parameters, the top row shows the highest correlation among all participants, while the bottom row shows the lowest correlation. Further, the left column shows the participant with the highest correlations among all parameters, while the right column shows the participant with the lowest correlations among all parameters.

Figure 6 detail (a) refers to the HR example shown in Figure 4 and detail (b) refer to the GSR example shown in Figure 5.

In summary, the overall pattern shown in Figure 6 confirms that both the type of the parameter measured and the individual parameters, such as skin characteristics of the participant, significantly influence the reliability of the measurements and thus the quality of further analysis.

#### 5.2.2. Cross-Correlation

**Figure 7:**
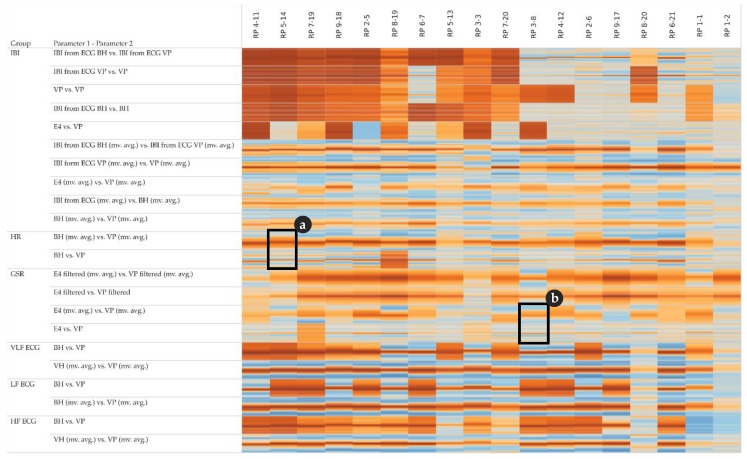
Cross-correlation matrix of pairs of parameters and participants; detail (**a**) complements Figure 4, detail (**b**) complements Figure 5; colour: orange indicates positive cross-correlation, blue indicates negative cross-correlation; a cell detail shows lags as small horizontal bars: lag −15 at top, and lag +15 at bottom.

In order to assess potential temporal shifts between the measurements of the same physiological parameter, we investigate the cross-correlation at different lags. The corresponding cross-correlation pattern in Figure 7 shows that some pairs of parameters (especially the top five rows of the IBI group) have a rather low variance among the lags and tend to correlate positively (i.e., the lags within a cell show rather homogeneous coefficients). Other pairs of parameters (especially the lower half of the IBI group) have a rather high variance among the lags, indicating positive correlations around lag 0 and negative correlations at lag +15 and −15, respectively.

Note that the cross-correlation matrix in Figure 7 is organized as follows: for each group of parameters, the top row shows the highest cross-correlations (i.e., lowest variance) among all participants, while the bottom row shows the lowest cross-correlation (i.e., highest variance). Further, the left column shows the participant with the highest cross-correlations among all parameters, while the right column shows the participant with the lowest cross-correlations among all parameters.

Figure 7 detail (a) refers to the HR example shown in Figure 4 and detail (b) refers to the GSR example shown in Figure 5.

#### 5.2.3. MINE Statistics

By using MINE statistics, we investigate relationships between and among parameter pairs of same as well as different type, for instance, GSR versus HR. In addition to the linear correlation coefficient of determination R^2^ (see Section 5.2.1), we use the Maximal Information Coefficient MIC to identify all functional relationships, also including linear ones as issued by R^2^. Although a functional relationship between certain combinations of parameters might be obvious (e.g., IBI [ms] = 60,000/HR [beats per hour]), we nonetheless include such combinations herein for reasons of confirmation.

**Figure 8:**
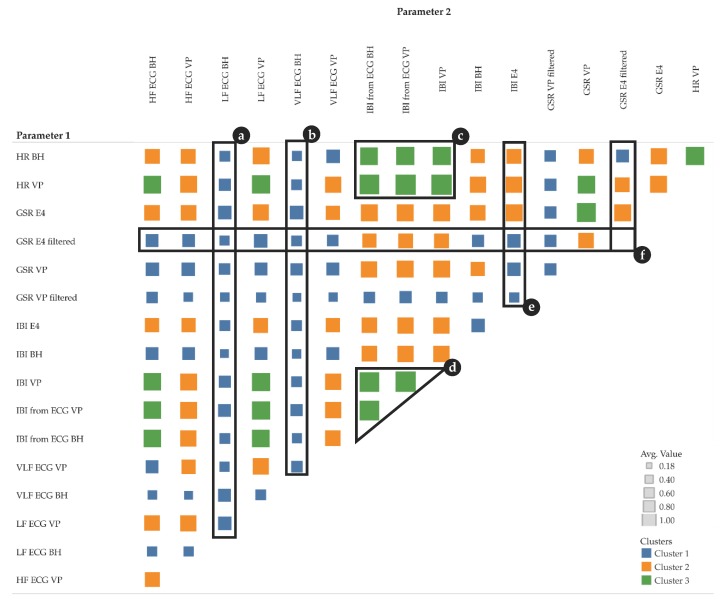
Maximum Information Coefficient (MIC) cluster matrix of pairs of parameters (size show averages among all participants, moving averaged versions only); colors: blue (cluster 1): low correlations; orange (cluster 2): moderate correlations; green (cluster 3): high correlations; symbol size in a matrix cell: average MIC among participants; (**a**–**f**) highlight special characteristics described in the text.

Figure 8 shows 3 k-means clusters of pairs of different parameter types. We tested with k [1,5] and chose 3 because the result is most intuitive—the clusters show low, moderate, and high correlations. Figure 8a–f highlights some particularly interesting parts of the clusters:

- a, b: LF and VLF derived from ECG measured by BioHarness show a rather low correlation with almost all other parameters
- c, d: IBI measurements, either directly measured or derived from ECG, show a rather high correlation with HR and with IBI from other sensors
- e, f: GSR measurements from VP (high- and low-pass filtered) show a rather low correlation with all other parameters; however, GSR measurements from E4 (high- and low-pass filtered) show a low to moderate correlation with all other parameters.

**Figure 9:**
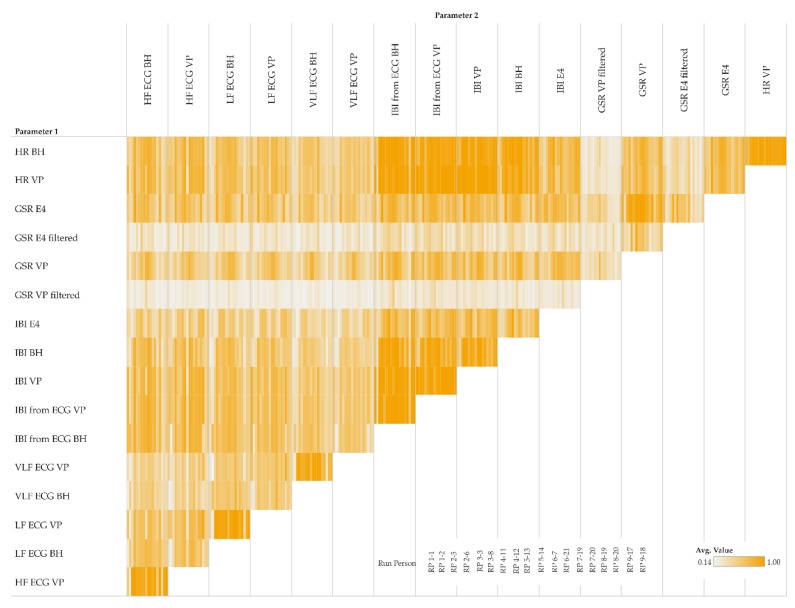
MIC participant-level matrix of pairs of parameters (moving averages only); details of a matrix cell show participants as small vertical bars (order of participants is shown in the legend).

When focusing on the level of individual participants, Figure 9 shows MIC correlations among pairs of different parameters. Within a single cell, the small vertical bars represent participants (one bar per participant). Figure 9 complements Figure 8 by adding participant information to the corresponding clusters.

**Figure 10:**
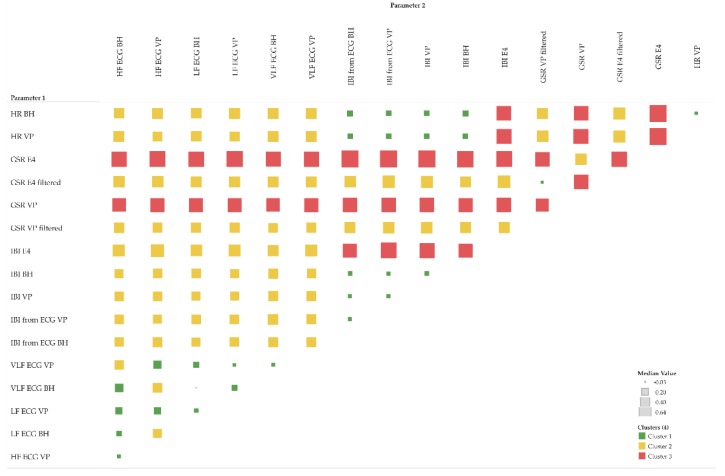
MIC–R^2^ cluster matrix of pairs of parameters (moving averages only); colors: green (cluster 1): “false” linear relationships; yellow (cluster 2): “true” linear relationships; red (cluster 3): functional but not linear relationships.

The MIC is used to quantify the strength of any functional relationships, i.e., including linear ones, while the R^2^ coefficient can only quantify linear relationships. By subtracting R^2^ from the MIC, we compute a measure of nonlinearity [36], which we use to identify the following three classes of relationships as shown in Figure 10:

- “false” linear relationships (R^2^ is not confirmed by MIC): MIC–R^2^ < 0
- “true” linear relationships (R^2^ is confirmed by MIC): MIC–R^2^ ~ 0
- functional but not linear relationships: MIC–R^2^ > 0

**Figure 11:**
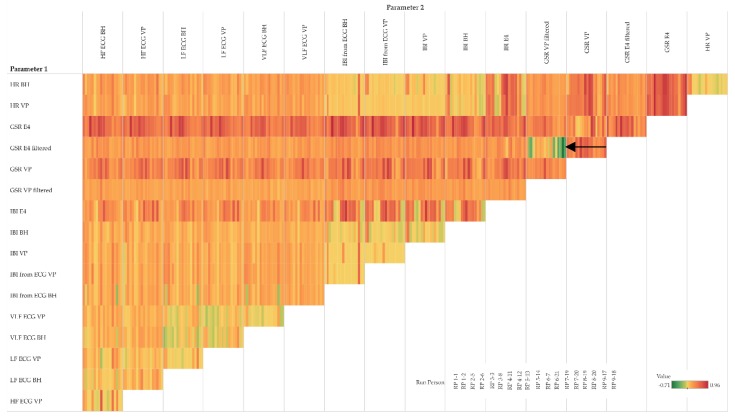
MIC–R^2^ individual matrix of pairs of parameters (moving averages only); details of a matrix cell show participants as small vertical bars (order of participants is shown in the legend); back arrow points to pairs of parameters with very weak association.

On the individual level, the MIC–R^2^ matrix shown in Figure 11 provides additional detail to the clustering view. Interestingly, GSR measurements from E4 and VP show rather strong functional but not linear relationships with almost all other parameters (see third and fifth row in Figure 10 and Figure 11). Particularly interesting is the relationship between GSR VP (filtered and moving averaged version, fourth row) and GSR E4 (filtered and moving averaged version, fifth-last column), which shows some highly negative values (see black arrow). These cases indicate a “false” linear relationship. For instance, participant RP 9–17: MIC 0.2 minus R^2^ 0.91 results in −0.71. In other words, the MIC does not confirm the highly linear relationship indicated by R^2^; in fact, the MIC indicates that there is almost no relationship. From a physiological point of view, this relationship might be obvious; however, the quantification of this relationship from a data-driven perspective is, to our best knowledge, novel.

#### 5.2.4. Fréchet Distance (Global and Local)

The Fréchet distance is a measure of how different two curves are from each other in terms of geometric structure [32]. Herein we use the Fréchet distance to measure the geometric similarity of two time series of a physiological parameter measured by different sensor platforms, one being professional and well-calibrated while the other is low-cost and wearable. In addition to the standard global Fréchet distance, we also compute local versions using a moving window approach.

**Figure 12:**
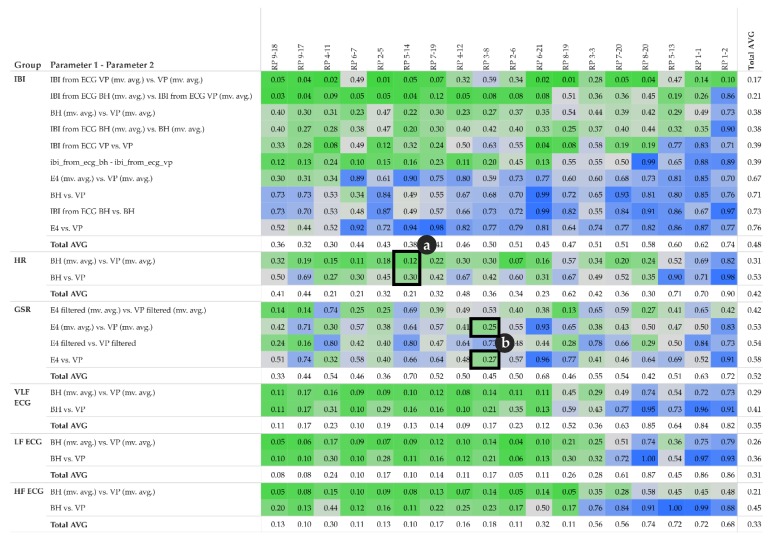
Global Fréchet distance matrix of pairs of parameter and participants; detail (**a**) complements Figure 4, detail (**b**) complements Figure 5; color: green indicates low distance thus high similarity, blue indicates high distance thus low similarity.

The global Fréchet distance matrix (Figure 12) shows two expectable general aspects. First, cardiac parameters such as IBI, HF, LF and VLF derived from ECG seem to be more similar than GSR. This is likely because, from a measuring point of view, ECG-related measurements are simply more robust than, for instance, GSR-related ones. Second, the moving averaged versions of the time series also tend to be more similar than their non-averaged counterparts, which include more local fluctuations. Figure 12 detail (a) refers to the HR example shown in Figure 4 and detail (b) refers to the GSR example shown in Figure 5. In both detail (a) and detail (b), the moving averaged time series causes a smoothing effect, thus indicating a higher similarity as compared to the original (non-smoothed) time series.

**Figure 13:**
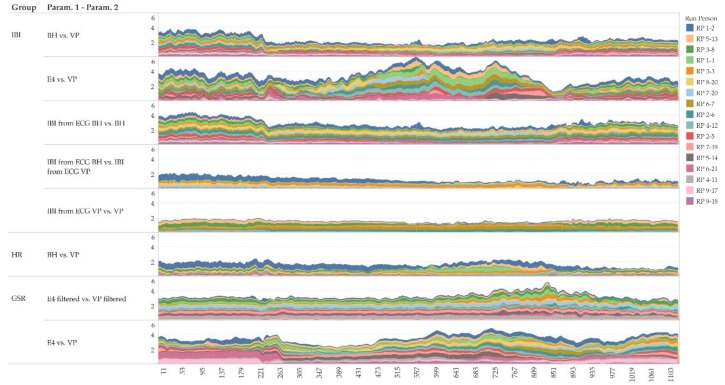
Local Fréchet distance of a moving time window (1 min) of selected pairs of parameters (inter beat interval IBI, heart rate HR, galvanic skin response GSR; moving averaged only).

In addition to the global geometric similarity, Figure 13 shows local similarity characteristics of the time series using a moving windows approach. The figure shows that the local Fréchet distance of a 1-min moving window indeed reveals differences in similarity at different intensities of physical activity (0–300 s: no activity; 301–900 s: cycling with increasing intensity; 901–1200 s: no activity–cool down; for details refer to Section 4.1). For instance, IBI derived from ECG tends to have a rather constant similarity over the entire measurement period (Figure 13, fourth row), and it tends to be more similar than IBI measured “directly” (Figure 13, first and second row).

#### 5.2.5. DTW Distance

**Figure 14:**
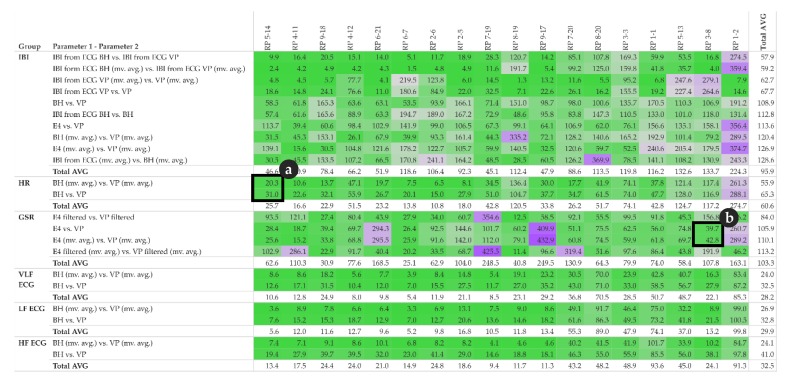
Dynamic Time Warping (DTW) distance matrix of pairs of parameter and participants; detail (**a**) complements Figure 4, detail (**b**) complements Figure 5; colour: green indicates low distance thus high similarity, purple indicates high distance thus low similarity.

The Dynamic Time Warping (DTW) distance is a measure typically used to assess the similarity of time series [50,51]. Simply speaking, DTW tries to optimize the alignment of one time series (test) with another (reference) by stretching or shrinking it in a non-linear fashion along its time axis. The overall distance is the sum of all distances between pairs of points. Identical time series have a distance of zero. Figure 14 shows the DTW distance between pairs of parameters and participants, detail (a) refers to the HR example shown in Figure 4 and detail (b) refer to the GSR example shown in Figure 5.

**Figure 15:**
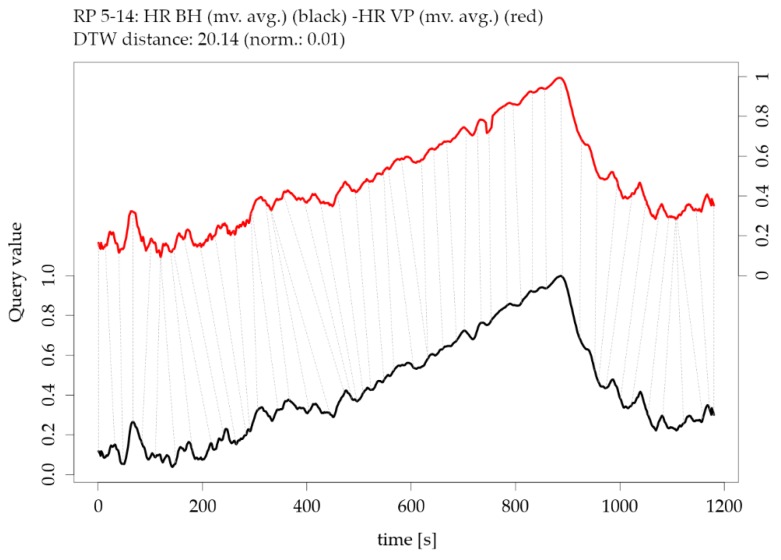
Illustration of the Dynamic Time Warping (DTW) distance between two parameters of participant RP 5-14: moving averaged version of heart rate HR from BioHarness BH versus moving averaged version of heart rate HR from VarioPort VP (note the offset of the two y-axes).

**Figure 16:**
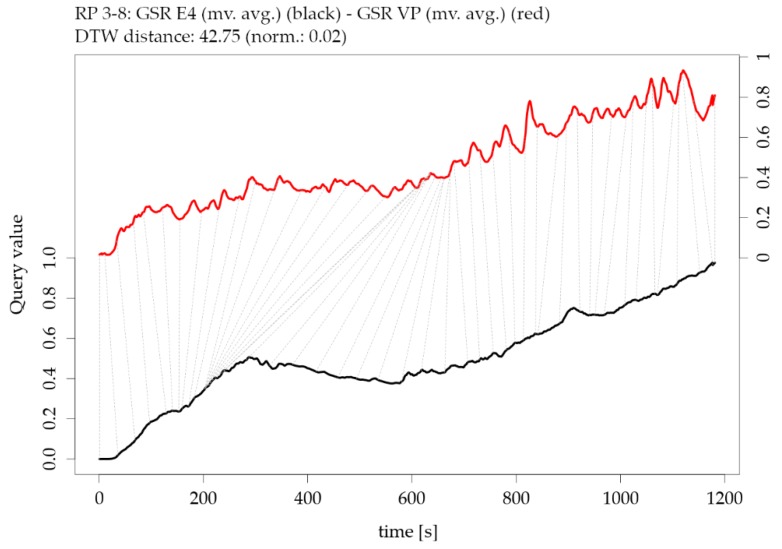
Illustration of the Dynamic Time Warping (DTW) distance between two parameters of participant RP 3-8: moving averaged version of galvanic skin response GSR from E4 versus moving averaged version of galvanic skin response GSR from VarioPort VP (note the offset of the two y-axes).

The global DTW distances from Figure 14 can be illustrated as an individual pairwise comparison of time series. For instance, Figure 15 shows an example of the DTW distance between two time series of one participant’s HR measurements, which are highly similar (low DTW distance). The corresponding exploratory plots of the HR example are shown in Figure 4**.** Figure 16 shows an example of two time series of GSR measurements with rather low similarity (high DTW distance); however, the overall trend is highly similar. The corresponding exploratory plots of the GSR example are shown in Figure 5.

## 6. Discussion and Limitations

Overall, the sensor benchmarking worked well, both from a standardized laboratory study and data acquisition viewpoint, as well as from the data analysis methodology perspective. The high correlations between the cardiovascular parameters HR and IBI were as expected because these parameters are comparably simple to measure through a range of methodologies (electrical, optical). The high correlations between the other ECG-derived measurements were a little more surprising because (1) ECG is measured through a multi-channel electric current-based system, which is a complex procedure; (2) the use of contact electrodes of the BioHarness sensor (in contrast to the sticky electrodes of high-quality sensors) may cause contact (and thus measurement-) problems; 3) ECG is measured at a very high frequency (at least 200 Hz), which is technologically challenging for low-cost wearables.

For GSR, our experiment resulted in lower, but still reasonable similarities, which may be caused by a number of factors like different measurement methods (sticky electrodes vs. plate electrode), and different placement of the sensors (hand palm vs. wrist), etc.

From a more general point of view, it is a known issue that low-cost wearable sensors tend to be prone to producing datasets that suffer from reduced data quality—even though we checked the appropriate positioning of the sensors before we started the exercise.

A particular issue arose with participant RP 1–2. As the results show, the measurements for this participant indicate low correlations for almost all physiological parameters. This may be due to problems with the contact between the electrodes and the skin, which may have been compromised by the person’s physical characteristics.

A vital part of the analysis is the visualisation of results on two complementary levels: First, on the individual level, the data from different sensors measuring the same physiological parameter at the same time on the same participant provide a direct comparison between the two-time series of interest. This enables reaching conclusions on the sensors’ measuring behaviour. Second, on the collective level, the consolidation of global metrics allows for comparing signals between participants. This further provides useful insights into the influence of the participants’ individual components (physical constitution, individual baseline level of skin conductance, etc.).

These complementary visualizations enable a flexible method of interpretation. For instance, it allows starting the interpretation on the individual level on a particular pair of physiological parameters of interest (e.g., HR of participant RP 5–14) using the corresponding exploratory plot as shown in Figure 4, then rolling-up using the R^2^ matrix (Figure 6a) together with the cross-correlation matrix (Figure 7a) and comparing the individual result between participants. Further, it allows checking whether that particular pair of parameters has a functional relationship and whether that relationship is stable among other participants by using the MIC–R^2^ individual matrix (Figure 11). The focus on a particular pair of parameters and participant can be continued to the similarity measures, namely the Fréchet distance and the DTW distance (Figure 12 and Figure 14). Another method of interpretation is to begin at the collective level using the MIC–R^2^ cluster matrix of pairs of parameters (Figure 10), then drilling-down on a specific parameter combination of interest using the MIC–R^2^ individual matrix (Figure 11) and contextualizing this matrix with the corresponding exploratory plots as shown in Figure 5.

This kind of visualisation provides the central advantage regarding the sensor benchmarking from a “big picture” view, i.e., to serve as a basis for visual analysis of the correlations between the measurements of one parameter as measured by two different sensors (each row in the matrix) and the correlations between the different parameters for a single participant (each column in the matrix). Furthermore, the matrix allows the simple assessment of each single cell to trace back particularities of each measurement to a test person, which makes it easier to single out anomalies that may be caused by usage errors, a user’s characteristics, single sensor failures, or violations of the benchmark protocol.

The cross-correlation analysis shows that groups of physiological parameters can be associated with different patterns of temporal shifts. As illustrated in Figure 7, the cross-correlation also varies between participants: from overall positive for IBI derived from ECG for more than 60% of the participants to highly positive at small lags and highly negative at larger lags at the heart rate variability parameters VLF, LF, and HF. Although the clocks of the sensors were synchronized right before the study, we observed a lag of 1–2 s between HR measurements (BH versus VP) and GSR measurements (E4 versus VP), as exemplarily shown in Figure 4a,c and Figure 5c,d, respectively. In all cases, the VP time series were “leading”, which may indicate that the response characteristics of the VP sensors are more sensitive compared to the other sensors.

Measuring the strength of association between pairs of physiological parameters was of particular interest. Herein, we contrasted the coefficient of determination R^2^ with the MIC (Figure 10 and Figure 11). In other words, we confronted a statistic that measures linear relationships against a statistic that measures all types of functional relationships, including linear ones, and thereby classified the relationship as ‘false linear’, ‘true linear’, or ‘functional but not linear’. The results are outstanding: on the one hand, some already expected linear relationships have been confirmed by a purely data-driven approach (for instance, relationships between IBI and VLF, LF, HF); on the other hand, some relationships that were expected to be linear are in fact not linear or functional. For instance, the relationships between GSR measured by E4 and GSR measured by VP (both filtered and moving averaged versions).

## 7. Conclusions and Future Work

In this paper, we performed a benchmark of two wearable physiological sensors (Zephyr BioHarness 3 and Empatica E4) by comparing their measurements (heart rate, inter-beat interval, and galvanic skin response, and derived heart rate variability parameters) to highly-calibrated high-end professional equipment. In our study, we used the measurements from 18 participants to compare the correlations (Pearson’s r), cross-correlations at different temporal lags from −15 sec to +15 sec, the (sub-)linearity of functional dependencies (MIC), the difference of two measurement time series with respect to their geometric structure (Fréchet distance), local time series similarities (moving window), and time series similarity with respect to their temporal alignment (DTW).

The results of our study show that the measured cardiovascular parameters yield very high similarities between the low-cost wearable and the calibrated professional sensors. Although cardiovascular parameters are simple to measure (technically and phenomenon-wise), the obtained similarities are remarkable. For GSR, our experiment resulted in lower similarities, which may be caused by a number of factors like different measurement methods, different placement of the sensors (hand palm vs. wrist), conduction characteristics between skin and sensor surface (use of electrolyte gel or not), and others. It should be noted that the use of isotonic electrolyte gel is a scientific standard for measurement of electrodermal activity [52] and was used with the Varioport GSR measure but not with the other devices.

We demonstrated that our methodological approach to quantify correlations and similarities on both the individual and the aggregated level can provide interesting insights into the relationships between and among physiological parameters. The many figures generated (only the most essential ones are presented in this paper) enable different points of view on the same data and thus a more holistic interpretation for the benchmark of physiological sensors. Our research contributes to such a holistic interpretation in two ways: 1) the confrontation of the coefficient of determination R^2^ against the Maximal Information Coefficient MIC, in particular, the classification of non-linear correlations, and 2) the quantification of the signals’ temporal and geometric similarity based on well-established distance metrics (DTW distance and Fréchet distance).

Our future work will focus on two main research challenges. First, to continue fine-tuning the methodology and integrate additional similarity measures, for instance, the Time Warp Edit Distance (TWED) [53]. Second, to evaluate the transferability of the methodology to other time series benchmarking challenges, not necessarily physiological measurements. In the long run we want to expand the methodology to the geospatial domain, i.e., integrating the location in addition to the timestamp and the measurement of mobile sensors. This approach will likely warrant an additional field study that addresses the suitability of measurement devices and measurement quality on moving subjects, e.g., persons riding a bicycle or walking, and relating sensor data to subjective experience self-report data.

## Figures and Tables

**Table 1:** Benchmarked sensors and physiological parameters of interest.

	VarioPort	Zephyr BioHarness 3	Empatica e4
	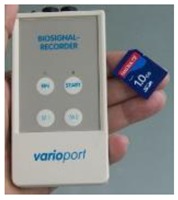	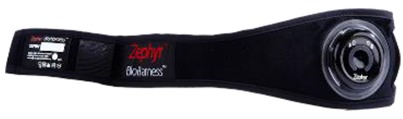	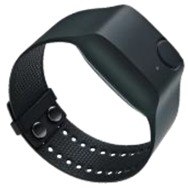
HR	X	X	–
IBI	X	X	X
ECG	X	X	–
GSR	X	–	X
